# Improved prediction of early cognitive impairment in multiple sclerosis combining blood and imaging biomarkers

**DOI:** 10.1093/braincomms/fcac153

**Published:** 2022-07-08

**Authors:** Tobias Brummer, Muthuraman Muthuraman, Falk Steffen, Timo Uphaus, Lena Minch, Maren Person, Frauke Zipp, Sergiu Groppa, Stefan Bittner, Vinzenz Fleischer

**Affiliations:** Department of Neurology, Focus Program Translational Neuroscience (FTN) and Immunotherapy (FZI), Rhine Main Neuroscience Network (rmn2), University Medical Center of the Johannes Gutenberg University Mainz, Langenbeckstr, 1, Mainz 55131, Germany; Department of Neurology, Focus Program Translational Neuroscience (FTN) and Immunotherapy (FZI), Rhine Main Neuroscience Network (rmn2), University Medical Center of the Johannes Gutenberg University Mainz, Langenbeckstr, 1, Mainz 55131, Germany; Department of Neurology, Focus Program Translational Neuroscience (FTN) and Immunotherapy (FZI), Rhine Main Neuroscience Network (rmn2), University Medical Center of the Johannes Gutenberg University Mainz, Langenbeckstr, 1, Mainz 55131, Germany; Department of Neurology, Focus Program Translational Neuroscience (FTN) and Immunotherapy (FZI), Rhine Main Neuroscience Network (rmn2), University Medical Center of the Johannes Gutenberg University Mainz, Langenbeckstr, 1, Mainz 55131, Germany; Department of Neurology, Focus Program Translational Neuroscience (FTN) and Immunotherapy (FZI), Rhine Main Neuroscience Network (rmn2), University Medical Center of the Johannes Gutenberg University Mainz, Langenbeckstr, 1, Mainz 55131, Germany; Department of Neurology, Focus Program Translational Neuroscience (FTN) and Immunotherapy (FZI), Rhine Main Neuroscience Network (rmn2), University Medical Center of the Johannes Gutenberg University Mainz, Langenbeckstr, 1, Mainz 55131, Germany; Department of Neurology, Focus Program Translational Neuroscience (FTN) and Immunotherapy (FZI), Rhine Main Neuroscience Network (rmn2), University Medical Center of the Johannes Gutenberg University Mainz, Langenbeckstr, 1, Mainz 55131, Germany; Department of Neurology, Focus Program Translational Neuroscience (FTN) and Immunotherapy (FZI), Rhine Main Neuroscience Network (rmn2), University Medical Center of the Johannes Gutenberg University Mainz, Langenbeckstr, 1, Mainz 55131, Germany; Department of Neurology, Focus Program Translational Neuroscience (FTN) and Immunotherapy (FZI), Rhine Main Neuroscience Network (rmn2), University Medical Center of the Johannes Gutenberg University Mainz, Langenbeckstr, 1, Mainz 55131, Germany; Department of Neurology, Focus Program Translational Neuroscience (FTN) and Immunotherapy (FZI), Rhine Main Neuroscience Network (rmn2), University Medical Center of the Johannes Gutenberg University Mainz, Langenbeckstr, 1, Mainz 55131, Germany

**Keywords:** multiple sclerosis, serum neurofilament, cognition, lesion volume, grey matter

## Abstract

Disability in multiple sclerosis is generally classified by sensory and motor symptoms, yet cognitive impairment has been identified as a frequent manifestation already in the early disease stages. Imaging- and more recently blood-based biomarkers have become increasingly important for understanding cognitive decline associated with multiple sclerosis. Thus, we sought to determine the prognostic utility of serum neurofilament light chain levels alone and in combination with MRI markers by examining their ability to predict cognitive impairment in early multiple sclerosis. A comprehensive and detailed assessment of 152 early multiple sclerosis patients (Expanded Disability Status Scale: 1.3 ± 1.2, mean age: 33.0 ± 10.0 years) was performed, which included serum neurofilament light chain measurement, MRI markers (i.e. T_2_-hyperintense lesion volume and grey matter volume) acquisition and completion of a set of cognitive tests (Symbol Digits Modalities Test, Paced Auditory Serial Addition Test, Verbal Learning and Memory Test) and mood questionnaires (Hospital Anxiety and Depression scale, Fatigue Scale for Motor and Cognitive Functions). Support vector regression, a branch of unsupervised machine learning, was applied to test serum neurofilament light chain and combination models of biomarkers for the prediction of neuropsychological test performance. The support vector regression results were validated in a replication cohort of 101 early multiple sclerosis patients (Expanded Disability Status Scale: 1.1 ± 1.2, mean age: 34.4 ± 10.6 years). Higher serum neurofilament light chain levels were associated with worse Symbol Digits Modalities Test scores after adjusting for age, sex Expanded Disability Status Scale, disease duration and disease-modifying therapy (B = −0.561; SE = 0.192; *P* = 0.004; 95% CI = −0.940 to −0.182). Besides this association, serum neurofilament light chain levels were not linked to any other cognitive or mood measures (all *P*-values > 0.05). The tripartite combination of serum neurofilament light chain levels, lesion volume and grey matter volume showed a cross-validated accuracy of 88.7% (90.8% in the replication cohort) in predicting Symbol Digits Modalities Test performance in the support vector regression approach, and outperformed each single biomarker (accuracy range: 68.6–75.6% and 68.9–77.8% in the replication cohort), as well as the dual biomarker combinations (accuracy range: 71.8–82.3% and 72.6–85.6% in the replication cohort). Taken together, early neuro-axonal loss reflects worse information processing speed, the key deficit underlying cognitive dysfunction in multiple sclerosis. Our findings demonstrate that combining blood and imaging measures improves the accuracy of predicting cognitive impairment, highlighting the clinical utility of cross-modal biomarkers in multiple sclerosis.

## Teaser

Brummer *et al.* report that a machine learning approach using a combination of blood and imaging measures improves the predictive accuracy of cognitive impairment in early multiple sclerosis. Their findings highlight the clinical utility of cross-modal biomarkers in multiple sclerosis patients who are likely to experience cognitive decline.

## Graphical Abstract

**Graphical abstract:**
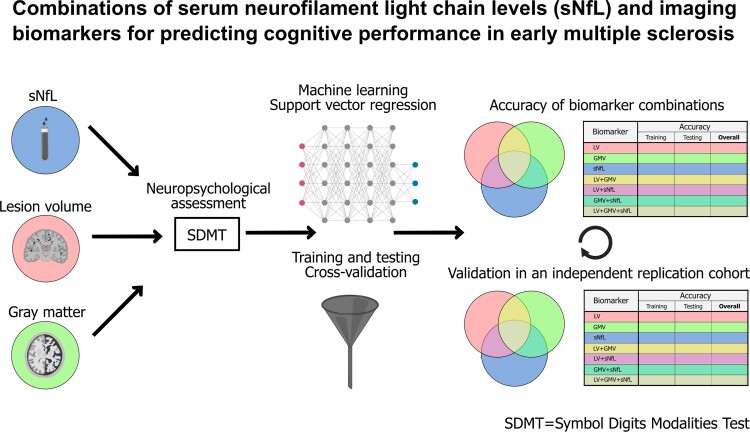

## Introduction

Disability in multiple sclerosis is generally classified by sensory and motor symptoms, yet cognitive impairment has been identified as a frequent manifestation already in the early disease stages.^1,2^ The most commonly affected cognitive domains include information processing speed, attention, and episodic memory, as well as impairments in executive function and verbal fluency.^3,4^ Deficits in these core domains have a detrimental influence on working ability and quality of life.^5,6^ Thus, monitoring cognitive decline in multiple sclerosis is crucial for individualized treatment decisions and development of new therapeutic paradigms.

Neuropsychological evaluation utilizes different test batteries to examine several cognitive domains and is thus able to give an in-depth characterization of patients’ cognitive performance. However, neuropsychological examinations are laborious, often limited to large facilities and thus not generally available in the clinical routine. Therefore, it is necessary to identify surrogate markers, which can be obtained by widely available methods and additionally serve as a biomarker of early cognitive impairment in multiple sclerosis patients. However, a substantial gap still exists between candidate biomarkers, validated biomarkers and clinically useful biomarkers in multiple sclerosis.^7^

MRI provides the most established biomarker for cognitive deficits in multiple sclerosis. In particular, conventional structural MRI metrics, like T_2_-hyperintense lesion volume (LV) and grey matter volume (GMV), have been proven to be reproducible and well-validated in predicting cognitive performance in multiple sclerosis.^8–11^

Besides these imaging markers with good spatial resolution, serum neurofilament light chain (sNfL) is an emerging fluid biomarker of neuro-axonal injury in many neurological conditions.^12–14^ In multiple sclerosis, sNfL levels are elevated during clinical relapses and correlate with an increase in T_2_-hyperintensive, as well as new gadolinium-enhancing lesions and brain atrophy.^15–20^ Thus, sNfL represents a marker for neuro-axonal damage and predicts disability progression.^14,15^ Nonetheless, little is known about the association of sNfL levels and cognitive decline in multiple sclerosis. The few recent studies have demonstrated mixed and inconsistent results depending on the investigated cohort. However, these studies may have been limited by their statistical power due to rather small sample sizes.^21–24^

Even though sNfL and MRI metrics have proven value in multiple sclerosis, there still is an urgent clinical need to be able to predict more accurately whether a patient will progress based on baseline measurements. Models combining blood- and imaging-based biomarkers that represent multiple aspects of multiple sclerosis pathology with key individual-level factors may improve prediction of cognitive performance.^25–27^ This is particularly true for complex and heterogeneous neurological disorders such as multiple sclerosis, in which a single marker might not be effective and only combinations of biomarkers can create signatures that are useful for improving the individual disease profiles.^7^ Hence, we here aimed to determine the utility of sNfL levels alone and in combination with MRI markers by examining their ability to predict cognitive impairment in early multiple sclerosis.

## Methods

### Participants

In total, 152 multiple sclerosis patients underwent MRI scans (details listed in the following context) and sNfL measurement at the outpatient clinic of the Department of Neurology, at the University Medical Center in Mainz (Germany) (Table 1). Of the 152 patients included, 34 had clinically isolated syndrome (CIS) with no dissemination in time, whereas the remaining 118 had relapsing–remitting multiple sclerosis (RRMS) as diagnosed according to the 2017 revised McDonald diagnostic criteria.^28^ The mean disease duration of all patients at time point of the first sNfL measurement (study entry) was 1.4 ± 2.2 years. Neuropsychological assessment (see details in the following context) took place within a mean of 9.3 ± 12.5 months after study inclusion. Each patient was clinically assessed by an experienced neurologist and their Expanded Disability Status Scale (EDSS) score was determined at study entry, along with demographic data. To confirm the reliability of the obtained results, we replicated the analyses by including an independent cohort of 101 early multiple sclerosis patients from our outpatient clinic (Table 1). Of the 101 patients included, 15 had CIS, whereas the remaining 86 had RRMS as diagnosed according to the 2017 revised McDonald diagnostic criteria.^28^ The mean disease duration of all patients until the first sNfL measurement was 1.0 ± 2.7 years. EDSS was determined at study entry; neuropsychological test assessment [in these patients, only the Symbol Digits Modalities Test (SDMT)] was performed within a mean of 2.8 ± 5.3 months after study inclusion. All cognitive evaluations were performed at least 30 days after a high-dose corticosteroid treatment, and no patient experienced a relapse within 30 days of cognitive assessment.

**Table 1:** Demographics and clinical characteristics

Demographics and clinical characteristics	Study cohort (*n* = 152)	Replication cohort (*n* = 101)	*P*-value	95% CI
Age: y, mean ± SD (median)	33.0 ± 10.0 (31)	34.4 ± 10.6 (34)	0.267^a^	−1.26 to 4.05
Sex, female: *n* (%)	107 (70.4)	70 (69.3)	0.853^b^	—
Disease course at diagnosis				
RRMS (%)	118 (77.6)	86 (85.1)	0.148^b^	—
CIS (%)	34 (22.4)	15 (14.9)		
Age at onset: y, mean, ± SD(median; 25th; 75th percentile)	30.9 ± 9.9 (29; 23; 39)	31.64 ± 9.84 (29; 24; 38)	0.673^a^	−1.95 to 3.01
Age at diagnosis: y, mean, ± SD (median; 25th; 75th percentile)	31.61 ± 10.0; (30; 24; 40)	33.42 ± 10.7 (32; 25; 39)	0.233^a^	−1.01 to 4.15
Disease duration: y, mean, ± SD (median; 25th; 75th percentile)	1.4 ± 2.2; (0.2; 0; 1.5)	1.0 ± 2.7 (0; 0; 1)	0.265^a^	−1.09 to 0.30
EDSS: mean ± SD (median; 25th; 75th percentile)	1.3 ± 1.2 (1; 0; 2)	1.1 ± 1.2 (1; 0; 2)	0.405^a^	−0.44 to 0.18
Treatment at time of MRI				
None (%)	43 (28.3)	33 (32.7)	0.456^b^	—
DMT (%)	109 (71.7)	68 (67.3)		
sNfL pg/ml: median (25th; 75th percentile)	9.6 (5.3; 16.8)	10.3 (6.8; 15.7)	0.633^a^	−0.08 to 0.13

^a^ Two-sided student’s t-test.

^b^ χ^2^ test.

95% CI = 95% confidence interval; sNfL = serum neurofilament light chain; RRMS = remitting–relapsing multiple sclerosis; CIS = clinical isolated syndrome; SD = standard deviation; EDSS = expanded disability scale score; DMT = disease-modifying therapy.

### Standard protocol approvals, registrations and patient consents

The study was approved by the local ethics committee (numbers: 2018–13622, 837.019.10); written informed consent was obtained from all patients.

### Clinical, cognitive and mood assessment

Cognitive performance was measured with the German Versions (if necessary) of the three neuropsychological instruments applied in multiple sclerosis studies: SDMT, Paced Auditory Serial Addition (PASAT) and the Verbal Learning and Memory Test (VLMT).^29–31^ For each subject, a *z*-score was calculated from normalized and normative values existing for each test, resulting in three test-specific *z*-scores per patient (SDMT score, VLMT score and PASAT score), corrected for age, sex and education according to normative values standardized on the basis of healthy individuals. No patient included in our cohorts had previously performed these cognitive assessment tests. In addition, patients were clinically assessed for affective parameters by means of the Hospital Anxiety and Depression Scale—which includes separate anxiety and depression subscales (HADS-A and HADS-D). Fatigue was measured with the common and established Fatigue Scale for Motor and Cognitive Functions (FSMCs)^32^ used in the diagnostic set-up and in the regular clinical follow-up. The FSMC is a patient self-reported questionnaire, which consists of 20 questions and evaluates the motor and cognitive components of fatigue. Currently, the FSMC tool is recommended for a multi-dimensional approach to reliably assess fatigue in patients with multiple sclerosis.^33^

### Serum neurofilament measurements

Serum samples were collected by attending physicians at the University Medical Center Mainz. Samples were processed at room temperature within 2 h: Serum samples were spun at 2000× g at room temperature for 10 min, aliquoted in polypropylene tubes and stored at −80°C. sNfL concentrations were measured as previously described.^13^ In brief, duplicates were measured by single-molecule array on a SiMoA HD-1 (Quanterix, USA) according to the manufacturer’s instructions using the NF-Light Advantage Kit (Quanterix, USA). The mean intra-assay coefficient of variation (CV) of duplicate determinations for concentration was 6.9%. Inter-assay CV was 2.5% for the low control mean with 3.7 pg/mL and 6.4% for the high control mean with 129.9 pg/mL. All measurements were performed in a blinded fashion with regard to diagnosis and clinical status of patients.

### MRI data acquisition

MRI data acquisition was performed as previously described.^34^ Structural MRI was performed on a 3-Tesla MRI scanner (Magnetom Tim Trio, Siemens, Germany) with a 32-channel receive-only head coil. In all patients, imaging was performed using a sagittal 3D T_1_-weighted magnetization-prepared rapid gradient echo sequence (TE/TI/TR = 2.52/900/1900 ms, flip angle = 9°, field of view = 256 × 256 mm2, matrix size = 256 × 256, slab thickness = 192 mm, voxel size = 1 × 1 × 1 mm^3^) and a sagittal 3D T_2_-weighted fluid-attenuated inversion recovery (FLAIR) sequence (TE/TI/TR = 388/1800/5000 ms, echo-train length = 848, field of view = 256 × 256 mm^2^, matrix size = 256 × 256, slab thickness = 192 mm, voxel size = 1 × 1 × 1 mm^3^). Major anatomical abnormalities were excluded by a clinician scientist blinded to the patient data based on the subject’s T_1_-weighted and FLAIR images of the whole brain.

### Quantification of white matter lesion and grey matter volume

The quantification of white matter (WM) lesion and grey matter (GM) volume was performed as previously described.^34^ In brief: The volumes of WM lesions were evaluated using the cross-sectional pipeline of the lesion segmentation toolbox,^35^ included in the Statistical Parametric Mapping (SPM8) software. Three-dimensional FLAIR images were coregistered to 3D T_1_-weighted images and bias-corrected. After partial volume estimation, lesion segmentation was performed with 20 different initial threshold values for the lesion growth algorithm.^35^ For each patient, the optimal threshold (κ value, dependent on image contrast) was determined and average values were calculated. A uniform κ value of 0.1 was applied in all patients to automatically estimate LV and filling of 3D T_1_-weighted images. Subsequently, the filled 3D T_1_-weighted images and the native 3D T_1_-weighted images were segmented into GM, WM and CSF and then normalized to Montreal Neurological Institute space. The quality of the segmentations was visually inspected to increase reliability.

### Statistic analysis

Statistical analysis was performed using SPSS 23 (SPSS, Chicago, IL, USA) and GraphPad Prism 5 software.

Summary statistics are presented as mean ± standard deviation (SD), median (25th and 75th percentile), or number (percentage), where applicable. Normal distribution was assessed by visual inspection and Kolmogorov–Smirnov test. The value of sNfL was log10-transformed to achieve approximate normality. sNfL values were then related to the respective test scores (SDMT, PASAT, VLMT, HADS-A, HADS-D and FSMC) by multiple linear regressions and adjusted for age, sex, EDSS, disease duration and disease-modifying therapy (DMT). MRI parameters (LV and GMV) were correlated with SDMT *z*-scores by multiple linear regressions adjusted for age, sex, EDSS, disease duration and DMT. Results from the linear regression model are expressed as regression coefficient (*B*) and standard error (SE). Demographics and clinical characteristics of the main and replication cohort were compared using two-sided student’s *t*-test and χ^2^/Fischer exact test. A *P*-value of <0.05 was considered statistically significant.

### Support vector machine regression model

The support vector machine (SVM) analysis is a machine learning tool for classification and regression. Although less popular than SVM, support vector regression (SVR) has been proven to be an effective tool in real-value function estimation.^36^ SVR represents a multiple regression method that is able to associate the observed and trained values and ultimately demonstrates the regression coefficient for the prediction accuracy. Here, we specifically applied SVR to test the significant results observed in our initial regression analysis, namely the association of sNfL, LV and GMV with SDMT, in order to test their combined predictive accuracies. We applied a data-driven regression model without explicitly stating a functional form indicating a non-parametric technique.^36^ The dependent variables were the respective SDMT *z*-scores and the independent variables were sNfL, LV and GMV. In brief: the algorithm looks for an optimally separating threshold between the respective datasets by maximizing the margin between classes’ closest points. The points located on the boundaries are the so-called support vectors, and the centre of the range represents the optimal separating threshold. In most cases, a linear separator is not optimal, thus a higher-dimensional space-projection needs to be performed where the data points become linearly interrelated. Owing to its good performance, we applied the radial basis function kernel for this projection. We used a grid search (min = 1; max = 10) to find the optimal input parameters, namely R (type of regression algorithm; 1 to 1000) and gamma (0.25). The selection was checked via 10-fold cross-validation by using 75% of the data for training of the algorithm and 25% for its testing. Cross-validation is a verification technique that evaluates the generalization ability of a model for an independent dataset.^36^ A soft-margin classifier of the calculated independent variables (sNfL, LV and GMV) was used for every parameter, and spurious correlations were weighted by a penalty constant, P. To optimize regression accuracy, we repeated the calculation for every regressor. The validation scheme was applied to assess whether the included independent variables (sNfL, LV and GMV) survived in the linear regression. For the SVR, we corrected for age, sex, EDSS, disease duration and DMT.

### Data availability

Restrictions apply to the availability of these data, which were used under licence for the present study and are therefore not publicly available. The raw data used in preparation of the figures and tables will be shared in anonymized format upon reasonable request by a qualified investigator for purposes of replicating procedures and results.

## Results

### Patient characteristics

An overview of the demographics and clinical characteristics of the investigated cohort is depicted in Table 1. The mean (± SD) age of the included 152 patients was 33.0 ± 10.0 years; 107 (70.4%) patients were female and 44 (29.6%) patients were male. The mean disease duration until the sNfL measurement was performed was 1.4 ± 2.2 years and the median disability (quantified with the EDSS score) was 1.0 (25th and 75th percentile: 0.0–2.0). Forty-three patients (28.3%) had no DMT at the time of inclusion, 109 (71.7%) received a DMT.

**Figure 1:**
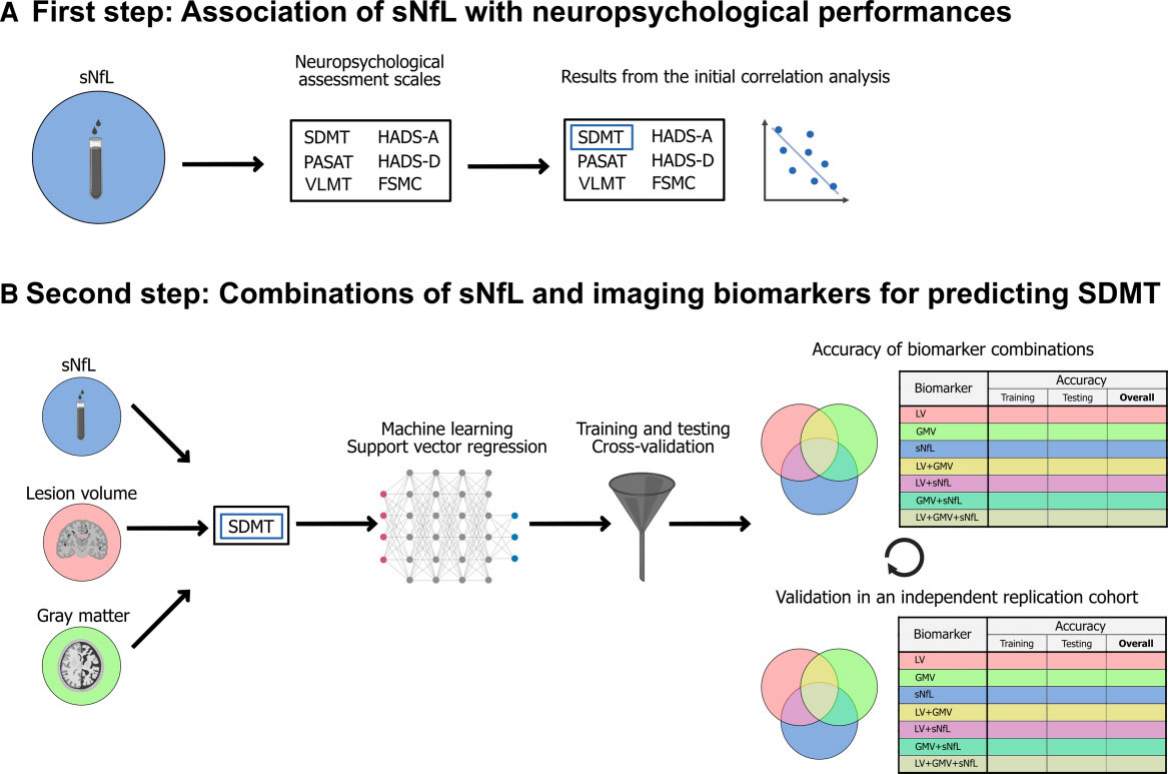
**Two-stage workflow.** (**A**) Association of sNfL with neuropsychological test performances. (**B**) Combinations of sNfL and imaging markers for the prediction of SDMT performance using a support vector regression approach. sNfL = serum neurofilament light chain; SDMT = Symbol Digits Modalities Test; PASAT = Paced Auditory Serial Addition Test; VLMT = verbal learning and memory test; HADS-A = Hospital Anxiety and Depression Scale (Anxiety); HADS-D = Hospital Anxiety and Depression Scale (Depression); FSMC = Fatigue Scale for Motor and Cognitive functions; LV = lesion volume; GMV = grey matter volume.

An overview of the demographics and clinical characteristics of the replication cohort is also depicted in Table 1. The mean ( ± SD) age of the included 101 patients was 34.4 ± 10.6; 70 (69.3%) patients were female and 31 (30.7%) patients were male. The mean disease duration until the sNfL measurement was performed was 1.0 ± 2.7 years and the median disability (quantified with the EDSS score) was 1.0 (25th and 75th percentile: 0.0–2.0). Thirty-three patients (32.7%) had no DMT at the time of inclusion, and 68 (67.3%) received a DMT. Appropriate statistical comparisons revealed no differences between the main and replication cohort (all *P*-values >0.05), thus showing comparability of our samples. An overview of our respective workflows is depicted in Fig. 1.

### Association of serum neurofilament light chain and MRI parameters with neuropsychological test scores

**Figure 2:**
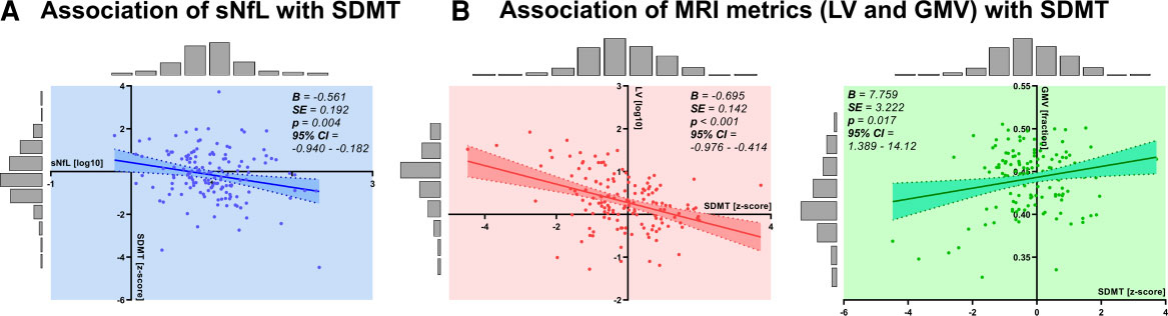
**Relationship between individual markers and SDMT performance.** (**A**) Associations of sNfL levels (log-transformed) with SDMT (z-scores). *N* = 149, *P* = 0.004; 95% CI = −0.940 to −0.182. (**B**) Association of MRI metrics [LV (log10-transformed) and GMV (fraction)] with SDMT (*z*-scores) each with the corresponding 95% confidence intervals. *N* = 152, LV: *P* < 0.001, 95% CI = −0.976 to −0.414; GMV: *P* = 0.017, 95% CI = 1.389–14.128. Histograms represent the data distribution of the cohorts. The *P*-value was adjusted for age, sex, EDSS, disease duration and DMT. B = Regression coefficient derived from linear regression; SE = Standard error derived from linear regression.

In our cohort of early multiple sclerosis patients, sNfL levels significantly correlated with patient performance in the information processing speed task after adjusting for age, sex, EDSS, disease duration and DMT (B = −0.561; SE = 0.192; *P = 0.004*; 95% CI = −0.940 to −0.182) (Fig. 2A, Table 2). However, sNfL levels were not significantly associated with the other investigated cognitive performance measures (PASAT: B = −0.248, SE = 0.204; *P = 0.225*, 95% CI = −0.065 to 0.155; VLMT: B = *−*0.069; SE = 0.182, *P* = 0.707, 95% CI = −0.428 to 0.291) or self-report questionnaires on fatigue (FSMC: B = 3.978; SE = 3.363; *P = 0.239*, 95% CI = −2.673 to 10.628), depression (HADS-D): B = 0.732; SE = 0.721; *P = 0.312,* 95% CI = −0.695 to 2.159 or anxiety (HADS-A): B = 0.011; SE = 0.803; *P* = 0.989, 95% CI = −1.578 to 1.600 (Table 2). Next, we sought to investigate the association of LV and GMV with SDMT performance. LV and GMV are commonly used structural MRI parameters, which have been associated with neurodegeneration in multiple sclerosis. Both LV (inversely) and GMV (positively) significantly correlated with the patients’ performance in SDMT and remained significant after multivariate regression for age, sex, EDSS, disease duration and DMT (LV: B = *−*0.695; SE = 0.142; *P < 0.*001, 95% CI = −0.976 to −0.414; GMV: B = 7.759; SE = 3.222; P = 0.017, 95% CI = 1.389–14.128) (Fig. 2B).

**Table 2:** Associations between sNfL and neuropsychological test performances

Test/questionnaire	B^a^ (*n*)	SE^b^ (*n*)	P-value^c^ (*n*)	95% CI^d^
Cognition				
SDMT	−0.561 (149)	0.192 (149)	0.004 (149)	−0.940 to 0.182
PASAT	−0.248 (137)	0.204 (137)	0.225 (137)	−0.651 to 0.155
VLMT	−0.069 (148)	0.182 (148)	0.707 (148)	−0.428 to 0.291
Depression and anxiety				
HADS-D	0.732 (130)	0.721 (130)	0.312 (130)	−0.695 to 2.159
HADS-A	0.011 (130)	0.803 (130)	0.989 (130)	−1.578 to 1.600
Fatigue				
FSMC	3.978 (142)	3.363 (142)	0.239 (142)	−2.673 to 10.628

^a^ Regression coefficient (B) derived from linear regression.

^b^ Standard error (SE) derived from linear regression.

^c^ *P*-value derived from linear regression adjusted for age, sex, EDSS, disease duration and DMT.

^d^ 95% confidence interval (CI) for B.

SDMT = Symbol Digits Modalities Test; PASAT = Paced Auditory Serial Addition Test; VLMT = verbal learning and memory test; HADS-A = Hospital Anxiety and Depression Scale (Anxiety); HADS-D = Hospital Anxiety and Depression Scale (Depression); FSMC = Fatigue Scale for Motor and Cognitive functions; DMT = disease-modifying therapy.

Taken together, sNfL, LV and GMV in patients with early multiple sclerosis significantly correlate with information processing speed (SDMT) test performance, whereas tests for memory, learning, affective parameters and fatigue did not show an association with sNfL levels.

### Prediction model of neuropsychological test performances

**Figure 3:**
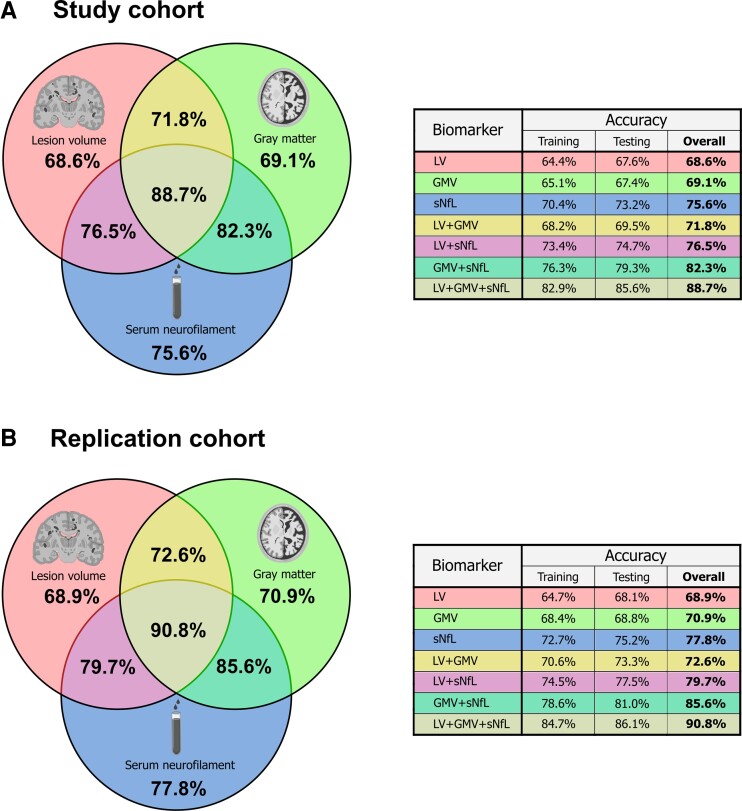
**Cross-validated prediction accuracies for single markers and combinations of markers.** Venn diagram depicting the results of the support vector regression analyses and the resultant cross-validated accuracies (**A**) in the study cohort and (**B**) in the replication cohort. LV = lesion volume; GMV = grey matter volume; sNfL = serum neurofilament light chain.

Next, we sought to create a prediction model to accurately predict SDMT performance by utilizing sNfL levels, LV and GMV. Thus, we employed a SVM regression analysis as described in the methods section. In the SVM approach, sNfL levels alone were 75.6% accurate at predicting SDMT scores, LV and GMV reached 68.6 and 69.1% accuracy, respectively (Fig. 3A). The combination of sNfL with either LV or GMV reached cross-validated accuracies of 76.5% (sNfL + LV) and 82.3% (sNfL + GMV), while LV and GMV combined were 71.8% accurate. The combination of all three parameters (sNfL + LV + GMV) achieved 88.7% cross-validated accuracy. To confirm the reliability of our results, we replicated the machine learning analysis in an independent cohort of 101 early multiple sclerosis patients (Table 1). In contrast to the main cohort, these patients had only undergone SDMT testing and were hence not eligible for inclusion in our main cohort with multiple neuropsychological tests. In the replication cohort, sNfL levels alone were 77.8% accurate at predicting SDMT scores, LV and GMV reached 68.9 and 70.9%, respectively (Fig. 3B). The combination of sNfL with either LV or GMV reached cross-validated accuracies of 79.7% (sNfL + LV) and 85.6% (sNfL + GMV), while LV and GMV combined were 72.6% accurate. The combination of all three parameters (sNfL + LV + GMV) achieved 90.8% cross-validated accuracy. Thus, by utilizing three biomarkers we obtained a robust prediction model for cognitive impairment in early multiple sclerosis.

## Discussion

In our study, we demonstrated that higher sNfL levels were associated with worse information processing speed in early multiple sclerosis. Besides this single cognitive domain parameter, sNfL levels were not linked to any other cognitive or mood measures. Both imaging markers, LV, as well as GMV were concurrently associated with patients’ information processing speed. With the aid of artificial intelligence, our unsupervised machine learning approach investigated the cross-validated accuracy in predicting SDMT using the combination of these three biomarkers. Here, the tripartite combination of sNfL, LV and GMV was highly accurate in predicting SDMT performance in the support vector regression, and outperformed each single biomarker as well as the dual biomarker combinations. Finally, these results were validated in an independent replication cohort using the same machine learning procedure. Hence, we showed that one blood-based and two imaging-based parameters combined provide a reliable and reproducible predictor of early information processing deficits in multiple sclerosis.

In a first step, we found that sNfL levels were inversely associated with SDMT performance and thus deficits in information processing speed. Using z-scores rather than a dichotomous classification allowed us to assume that the association of sNfL with SDMT was more properly represented as a continuum. Other cognitive tests, examining verbal learning and short-term memory (through the VLMT), auditory information processing, working memory, and divided attention (through the PASAT) and affective parameters (HADS-A and -D) did not show a significant association with sNfL. The lack of an association of sNfL with the other neuropsychological parameters seems to challenge the notion that slowed processing speed always underlie memory difficulties or anxiety and depression in multiple sclerosis.^37^ Even though PASAT revealed a trend towards an association with sNfL, this relationship failed to reach statistical significance. Despite also measuring information processing speed, PASAT focuses on auditory information processing, has proven less reliable than SDMT and is often considered too difficult, especially in progressive disease stages.^38^ Thus, in clinical as well as research practices, PASAT has largely been replaced by SDMT.

Previous studies have demonstrated inconsistent results regarding the association of sNfL and SDMT in multiple sclerosis,^21–24^ which may be related to sample sizes or composition of cohorts (inclusion of patients with CIS, RRMS and also progressive forms). Our cohort consisted of CIS or early RRMS patients with comparably short disease durations, thus reinforcing the notion that cognitive impairment is not limited to later or progressive disease stages but can be present from the onset. Nevertheless, our approach might also be applicable for patients in more advanced stages of the disease. However, the plateauing relationship between T_2_-lesion burden and clinical disability in the later disease stages needs to be taken into account.^39^ Since one part of our tripartite model relies on LV, it would also require further validation in a progressive cohort.

The SDMT has become a frequently investigated tertiary and exploratory outcome in clinical trials in recent years^6^ and efforts are underway to further adjust the EDSS for cognition.^5^ The here observed association of SDMT with sNfL levels suggest a close connection between cognitive processing and neuro-axonal damage. However, it has to be taken into account that the SDMT is a sensitive but non-specific parameter. While it emphasises information processing speed, multiple sclerosis patients’ general cognitive performance also depend on cognitive domains such as working memory, short- and long-term memory, paired-associate learning, and visual scanning. Hence, pre-existing deficits in these categories may confound SDMT performances, although this is rather unlikely in our early multiple sclerosis cohort. However, assessing the premorbid intelligence as a proxy for the participant’s cognitive reserve would have been valuable. Besides SDMT, sNfL has previously also been associated with disease activity and EDSS progression in multiple sclerosis,^15^ yet in our cohort we did not find such correlation. This may be due to our cohorts’ early disease stages and the overall low EDSS scores.

The underlying neurobiological basis of our findings may be that neuro-axonal damage (increased sNfL) particularly disturbs interconnected cognitive neuronal networks—which are crucially involved in information processing (diminished SDMT performance)—while leaving sensory and motor systems (EDSS functional systems) mostly unaffected.^40^ Alternatively, cognitive measures, such as SDMT, may have a higher sensitivity to detect variance in multiple sclerosis-related disability not encompassed by physical measures, especially in our early multiple sclerosis cohort.^41^

Based on the multiple regression analyses, we evaluated next whether conventional MRI biomarkers of inflammation (i.e. LV) and neurodegeneration (i.e. GMV) were likewise associated with SDMT performance. This should serve as a cross-modality validation given that SDMT has been shown to correlate with both MRI measures in a large body of studies.^11,42,43^ In line with these studies, our results showed that—in addition to sNfL—deficits in information processing speed were associated with LV and GMV. Previous studies have also demonstrated an association of regional volumetric measures (deep GMV, mesial temporal cortex, and neocortex, as well as total WM lesions) with different domains of cognitive impairment and neuropsychological test performances.^8–10,43^ Of the investigated MRI measures, GMV was shown to be the most reliable marker in predicting cognitive deficits in multiple sclerosis patients.^11^ However, the prediction of cognitive decline on an individual patient’s level has remained challenging on the basis of MRI features alone.^11,42^ Some previous attempts to combine MRI biomarkers to identify multiple sclerosis patients at risk of cognitive impairment have yielded promising results.^25,44^ In our study, we sought to increase the prediction accuracy by combining the two aforementioned MRI parameters with sNfL in a unsupervised machine learning approach (namely SVM) based on the idea that algorithms can learn from data and identify patterns with little human intervention.

Juxtaposing the three biomarkers for cognitive impairment, our machine learning approach revealed that sNfL achieved the highest cross-validated accuracy in predicting SDMT performance—in both the study and replication cohorts. This might be explained by a rapid disruption of neuro-axonal structures through inflammatory and demyelinating lesions promptly leading to higher sNfL levels and clinically to increased neuronal dysfunction,^14,15,45^ which manifests in cognitive impairment. GMV, however, probably displays time-delayed structural changes,^46^ which are not as prominent in our early multiple sclerosis cohort.

All double combinations achieved higher accuracies in predicting SDMT than the single measures alone. This pattern was successfully proven in the replication cohort. And, finally, the tripartite combination of all three potential predictors solidly outperformed the single and double predictors. Hence, by applying artificial intelligence, we were not only able to predict patients’ SDMT performances, but further demonstrated that the combination of sNfL, LV and GMV increased the predictive accuracy from 68.6 to 75.6% for the individual parameters to a cumulative accuracy of 88.7% in the main cohort and even 90.8% in the replication cohort. Thus, it is feasible to predict SDMT performance, by utilizing widely accessible surrogate parameters (MRI and sNfL), without entirely relying on neuropsychological testing. In this way—and towards a potential clinical application—our model might provide a surrogate screening tool for cognitive decline, whereby multiple sclerosis patients at risk for cognitive impairment may be selected for in-depth neuropsychological testing.

Accurate and neurobiologically plausible biomarkers can improve diagnostics, predict disease outcomes and enable monitoring of disease progression in many disorders,^12^ yet the discovery and clinical implementation of such biomarkers has been especially challenging in complex neurological conditions, such as multiple sclerosis. Our findings now reinforce the notion that if a single marker is too vague to forecast cognitive decline, the combination of multiple biomarkers may represent a robust and reproducible solution.

Recently, such an approach has been applied with the aid of molecular and MRI biomarkers to gain insight into brain volume loss after immunoablative autologous haematopoietic stem cell transplantation in multiple sclerosis.^26^ Another application field is neurodegenerative disorders, like Alzheimer’s disease, where a combination of CSF-biomarkers (Aβ_40–42_ and *P*-tau) and imaging parameters (hippocampal, temporo-parietal atrophy and amyloid-PET) has greatly improved diagnostic accuracy in recent years.^47,48^

Our study has several limitations. The cross-sectional design does not allow us to describe the time-dependent association of sNfL with cognitive decline in multiple sclerosis. In line with this, our study demonstrates a statistical prediction neglecting any meaning of time. Although we do not predict longitudinal cognitive performance, our model highlights the possibility to use the combination of blood and imaging biomarkers as a surrogate marker for cognitive impairment in multiple sclerosis. In addition, sNfL measurements at single time points can be subject to fluctuations and are therefore sub-optimal due to lack of intra-individual reproduction.^49^ Hence, studies are needed to identify variables independent of disease-related factors that modify sNfL levels. In this context, one recent study observed a correlation with body mass index and blood volume suggesting that potential sNfL associations might change if corrections for both measures are included.^50^ Another limitation of our study is the interval of about 9 months between study inclusion and neuropsychological testing. Finally, data were collected as part of the clinical routine with patients receiving different DMTs. However, our results did not change after correcting for DMT versus no DMT.

In conclusion, early neuro-axonal loss measured by sNfL correlates with worse information processing speed, the key deficit underlying cognitive dysfunction in multiple sclerosis. Combining blood and imaging measures improved the predictive, cross-validated accuracy of cognitive impairment, highlighting the clinical utility of cross-modal biomarkers in multiple sclerosis. Prognostic models based on combinations of biomarkers such as the one introduced here may improve the identification of multiple sclerosis patients who are likely to experience cognitive decline.

## Abbreviations

CIS =: clinically isolated syndrome
DMT =: disease-modifying therapy
EDSS =: Expanded Disability Status Scale
FSMC =: Fatigue Scale for Motor and Cognitive Functions
GMV =: grey matter volume
HADS =: Hospital Anxiety and Depression scale
LV =: lesion volume
PASAT =: Paced Auditory Serial Addition Test
RRMS =: relapsing–remitting multiple sclerosis
SDMT =: Symbol Digits Modalities Test
SiMoA =: single-molecule array
sNfL =: serum neurofilament light chain
SVR =: support vector regression
VLMT =: verbal learning and memory test
